# Thermal Reduction of MoO_3_ Particles and Formation of MoO_2_ Nanosheets Monitored by In Situ Transmission Electron Microscopy

**DOI:** 10.1021/acs.jpcc.3c05159

**Published:** 2023-10-26

**Authors:** Xiaodan Chen, Roos M. de Boer, Ali Kosari, Heleen van Gog, Marijn A. van Huis

**Affiliations:** † Soft Condensed Matter, Debye Institute for Nanomaterials Science, Utrecht University, Princetonplein 5, 3584 CC Utrecht, The Netherlands; ‡ Electron Microscopy Centre, Utrecht University, Universiteitsweg 99, 3584 CG Utrecht, The Netherlands; § Nanostructured Materials and Interfaces, Zernike Institute for Advanced Materials, University of Groningen, Nijenborgh 4, 9747 AG Groningen, The Netherlands

## Abstract

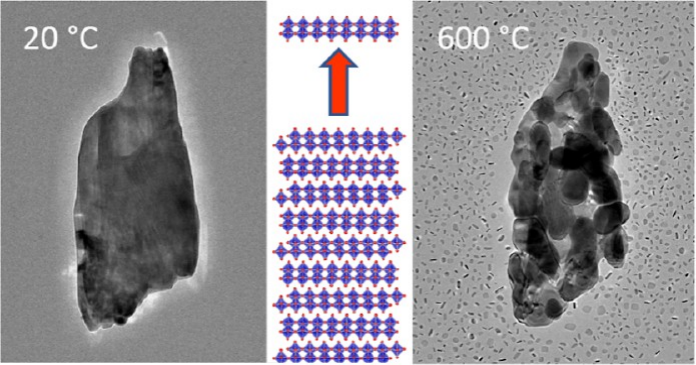

Nanoscale forms of molybdenum trioxide have found widespread use in optoelectronic, sensing, and battery applications. Here, we investigate the thermal evolution of micrometer-sized molybdenum trioxide particles during in situ heating in vacuum using transmission electron microscopy and observed drastic structural and chemical changes that are strongly dependent on the heating rate. Rapid heating (flash heating) of MoO_3_ particles to a temperature of 600 °C resulted in large-scale formation of MoO_2_(001) nanosheets that were formed in a wide area around the reducing MoO_3_ particles, within a few minutes of time frame. In contrast, when heated more gently, the initially single-crystal MoO_3_ particles were reduced into hollow nanostructures with polycrystalline MoO_2_ shells. Using density functional theory calculations employing the DFT-D3 functional, the surface energy of MoO_3_(010) was calculated to be 0.187 J m^–2^, and the activation energy for exfoliation of the van der Waals bonded MoO_3_ (010) layers was calculated to be 0.478 J m^–2^. Ab initio molecular dynamics simulations show strong fluctuations in the distance between the (010) layers, where thermal vibrations lead to additional separations of up to 1.8 Å at 600 °C. This study shows efficient pathways for the generation of either MoO_2_ nanosheets or hollow MoO_2_ nanostructures with very high effective surface areas beneficial for applications.

## Introduction

Molybdenum oxides are versatile materials occurring in various compositions and structural polymorphs and have applications in many fields. They are used in, among others, batteries,^1,2^ electrochromic materials,^3^ gas sensors,^4,5^ and OLEDs.^6,7^ The molybdenum trioxide (MoO_3_) is a particularly interesting member of the molybdenum oxide family, as it has a layered structure and is often fabricated in planar morphologies such as thin films, 2D nanosheets, or flakes.^1,5,8^

**Figure 1:**
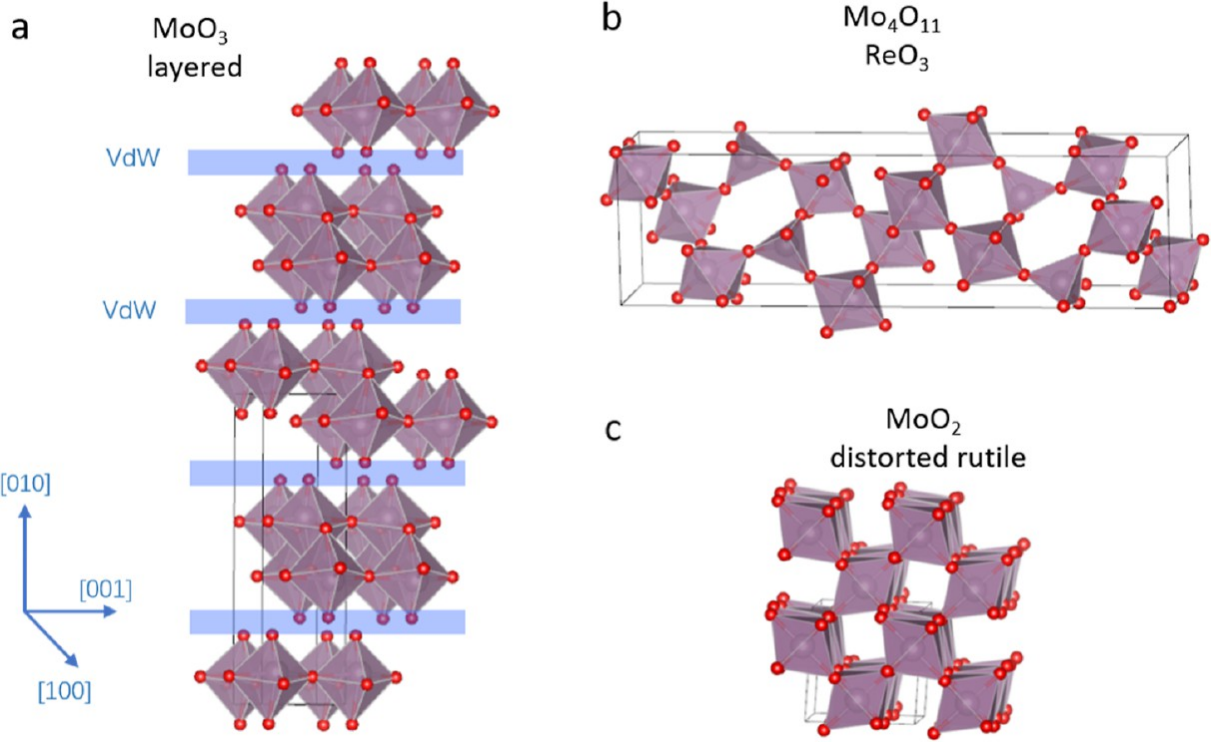
Crystal structure of (a) orthorhombic MoO_3_ with vdW-bonded layers, (b) Mo_4_O_11_, and (c) monoclinic MoO_2_. Purple spheres denote Mo atoms, while red spheres denote oxygen atoms. Crystallographic details are given in Table 1.

MoO_3_ has an orthorhombic structure with layers of distorted MoO_6_ octahedra. The layers are bound along the [010] direction by van der Waals (vdW) interactions. Within one layer, octahedra are corner shared along the [100] and [001] directions. In contrast, molybdenum dioxide MoO_2_ has a monoclinic distorted rutile-type structure. Here, the MoO_6_ octahedra share edges along the [001] direction. Also phases with intermediate compositions of MoO_3–*x*_ (0 < *x* < 1) have been observed. With the reduction of MoO_3_ in an oxygen-deficient environment, there are many MoO_3–*x*_ (0 < *x* < 1) phases occurring with a ReO_3_-type structure, like Mo_9_O_26_, Mo_8_O_23_, and Mo_4_O_11_. These phases are described as Magnéli series with composition Mo_*n*_O_3*n*–1_.^9−11^ In 1969, Bursill^12^ reported the thermal decomposition of MoO_3_ induced by beam heating and in years thereafter, the structures of MoO_3_ and of ReO_3_ types were also investigated.^13,14^ The crystal structure information and schematic structures of MoO_3_, MoO_2_, and Mo_4_O_11_ are listed in Table 1 and are displayed in Figure 1. Other lattice spacings of the three oxides are given in Tables S1–S3 of the Supporting Information.

**Table 1:** Structural Details of MoO_*x*_ Phases^11,15,16^

	MoO_3_	MoO_2_	Mo_4_O_11_
structure	orthorhombic	monoclinic	orthorhombic
space group	*Pbnm*	*P*2_1_/*c*	*Pn*2_1_*a*
lattice parameter (Å)	*a* = 3.966; *b* = 13.88; *c* = 3.703	*a* = 5.608; *b* = 4.842; *c* = 5.517	*a* = 24.400; *b* = 5.450; *c* = 6.723

The reduction process of MoO_3_ to MoO_2_ has been investigated in various studies.^17−26^ Mainly two types of reduction processes were reported. The first one is the one-step process, in which MoO_3_ reduces directly to MoO_2_ without any intermediate phases. The second one is a two-step process in which Mo_4_O_11_ is also involved. In the reports in which Mo_4_O_11_ was observed, various mechanisms were proposed. In 1978, Burch^27^ first found the formation of Mo_4_O_11_ during the reduction. Ressler et al.^28^ investigated the reduction with H_2_ during annealing and reported that the reduction process depended on the temperature. When the temperature was below 425 °C, MoO_3_ reduced to MoO_2_ directly. Otherwise, the Mo_4_O_11_ was formed in a parallel reaction. Lalik^29^ proposed an autocatalytic comproportionation kinetics model which means that the MoO_2_ first formed on the surface of MoO_3_ after which it reacted with the remainder of MoO_3_ and formed Mo_4_O_11_. Dang et al.^30^ reported a consecutive mechanism in which the transformation of MoO_3_ into Mo_4_O_11_ and of Mo_4_O_11_ into MoO_2_ proceeded simultaneously. The in situ X-ray photoelectron spectroscopy study by Garland et al. suggested that several Magnéli phases are present during the reduction from MoO_3_ to MoO_2_.^31^

**Figure 2:**
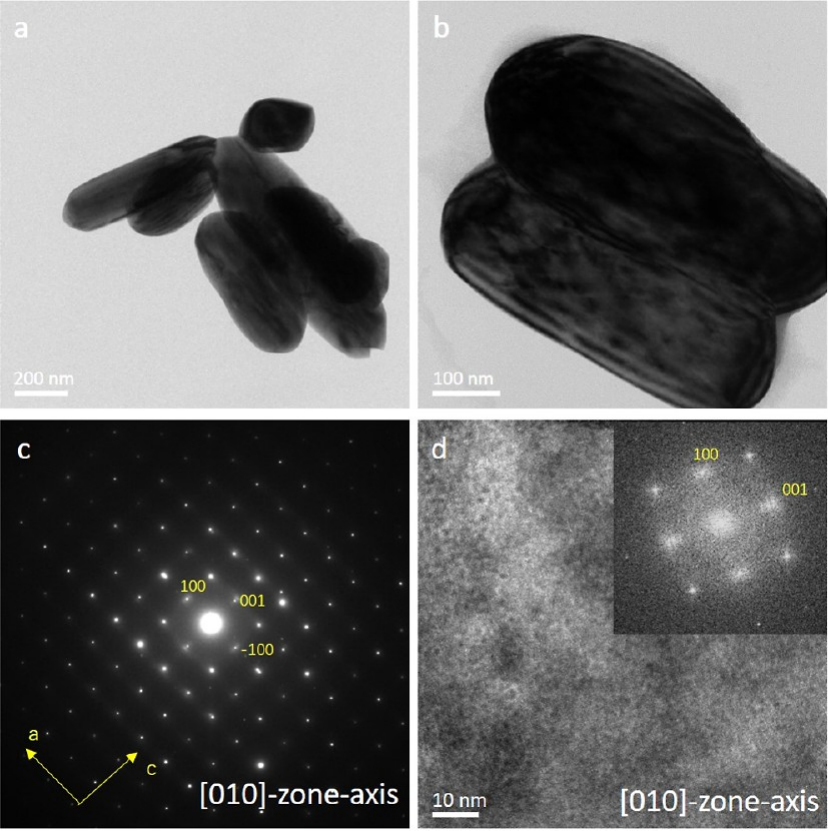
(a,b) Bright-field TEM images of MoO_3_ particles at room temperature; (c) diffraction pattern; and (d) high-resolution TEM image with the FFT in the inset. (c) and (d) are both taken in the [010]-zone axis.

In this study, the thermal behavior of the MoO_3_ particles was investigated. Figure 2 shows transmission electron microscopy (TEM) images and electron diffraction (ED) patterns of the pristine MoO_3_ particles that are the starting points of this study. Their typical morphology can be seen from the scanning electron microscopy (SEM) images in Figure S1 of the Supporting Information. These particles were heated under high vacuum conditions, acting as an oxygen-poor environment. When heated at a rapid pace, a fast exfoliation of nanoflakes from the larger MoO_3_ particles was observed. The resulting nanoflakes were found to be reduced to lower oxidation states. When heated gently, however, exfoliation takes place more slowly and the particles were reduced to hollow shell structures with the MoO_2_ phase. Complementary to these experimental investigations, density functional theory (DFT) calculations were used to investigate the energetics of the MoO_3_ nanoparticle reduction. DFT is a quantum mechanical computational method that uses the Schrödinger equation to perform calculations on molecules and crystal structures. Using DFT, the stability of the different molybdenum oxide phases is assessed and the reaction energies calculated. Furthermore, ab initio molecular dynamics (AIMD) simulations were performed to investigate the effects of thermal vibrations on the stability of the layered MoO_3_ structure.

## Methods

### TEM and SEM Investigations

The MoO_3_ particles were purchased from Sigma-Aldrich and had a broad size distribution. Most of the particles are in the size range 200 nm to 1 μm. Bright-field TEM images and selected area diffraction patterns (SADPs) were conducted using a FEI TalosF200X TEM operating at 200 kV. High-angle annular dark field scanning electron microscopy (HAADF–STEM) images were taken using a Thermo Fisher Scientific Spectra300 operating at 300 kV. The SEM images, as shown in Figure S1, are recorded using a TFS Helios Nanolab G3 operating at 30 kV using a secondary electron (SE) detector. The in situ heating in the TEM was conducted using a dedicated DENSsolutions heating holder. The specimens were prepared by drop casting the MoO_3_ solution onto a DENSsolutions MEMS heating chip with windows covered by a SiN membrane for observation. The chip was then mounted on the holder. The MoO_3_ specimens were first heated from 20 to 600 °C with 100 °C increments. In a second set of heating experiments, the specimens were heated from 20 to 400 °C with 100 °C increments but more gently with smaller increments of 25 °C when raising the temperature further from 400 °C to a maximum of 700 °C. Plots of typical heating profiles are shown in Figure S2. Great care was taken to exclude any influence of the electron beam on the observations. The electron beam illuminates only a very small part of the sample area. The field of view was changed frequently to verify that in areas previously not exposed to the electron beam, the particles underwent the same thermal evolution. Electron energy loss spectrometry (EELS) measurements were performed on pristine MoO_3_ particles (before heating) and on the MoO_2_ nanosheets that were formed after heating. The EELS measurements were performed in a Spectra300 TEM operating at 300 kV in the STEM mode, using a Gatan Continuum HR/1066 spectrometer.

### DFT Calculations

To obtain more insights into the energetics of the observed transformations, plane-wave DFT calculations were conducted using the VASP code.^32,33^ The energy cutoff for the wave functions and the density of the *k*-mesh were tested on the unit cell of the MoO_3_ structure in order to ascertain energy convergence within 0.5 meV/atom. The cutoff energy for the wave functions was set to 800 eV and the cutoff energy for the augmentation functions to 1120 eV while the *k*-mesh was set at 6 × 2 × 6. For MoO_2_ and Mo_4_O_11_, the *k*-mesh was rescaled to the lattice parameters to have a similar density of the *k*-mesh, yielding a *k*-mesh of 6 × 8 × 6 for MoO_2_ and a *k*-mesh of 2 × 8 × 6 for Mo_4_O_11_. The calculations were performed using the general gradient approximation (GGA) employing the exchange–correlation functional of Perdew, Burke, and Ernzerhof (PBE).^34,35^ To account for the vdW interactions in the layered MoO_3_ structure, as displayed in Figure 1, two other functionals were tested as well to find which one gives the best agreement with experimental values: the DFT-D3^36^ functional, which adds a vdW interaction to the GGA–PBE functional, and the optB88-vdW^37−40^ functional, which is a nonlocal exchange–correlation functional that accounts for dispersion interactions and is optimized for the correlation part. For each functional, the cell was relaxed with the optimal energy cutoff and *k*-mesh settings. The lattice parameters were compared to experimental values,^11,40^ and an overview of the results is given in Table 2.

**Table 2:** DFT-Calculated Lattice Parameters for MoO_3_, Mo_4_O_11_, and MoO_2_ for Three Different Functionals Compared to the Experimental Values^a^

	functional	*a* (Å)	Δ*a* (%)	*b* (Å)	Δ*b* (%)	*c* (Å)	Δ*c* (%)
MoO_3_	experimental	3.962		13.856		3.698	
	GGA-PBE	3.94	0.58	15.85	14.4	3.69	0.28
	DFT-D3	3.93	0.87	14.35	3.59	3.69	0.31
	OptB88-vdW	3.91	1.25	14.07	1.57	3.71	1.09
Mo_4_O_11_	experimental	24.29		5.457		6.752	
	GGA-PBE	24.73	1.83	5.52	1.16	6.81	0.89
	DFT-D3	24.71	1.72	5.52	1.16	6.80	0.74
	OptB88-vdW	24.66	1.52	5.49	0.65	6.78	0.42
MoO_2_	experimental	5.608		4.842		5.517	
	GGA-PBE	5.39	3.93	4.90	1.15	5.50	0.37
	DFT-D3	5.37	4.28	4.88	0.84	5.48	0.71
	OptB88-vdW	5.40	3.75	4.89	1.07	5.48	0.61

a The deviations of the calculated values from the experimental values are given in percentages.

For MoO_3_, the GGA–PBE functional performs poorly in predicting the *b* lattice parameter, which is in the layer stacking direction, with a deviation from the experimental value by as much as 14%. The DFT-D3 and optB88-vdW functionals give great improvements compared to the GGA–PBE functional, deviating in this particular lattice parameter by 3.6 and 1.6%, respectively. The optB88-vdW functional predicts best the lattice parameters compared with the experimental values. For MoO_2_ and Mo_4_O_11_, the best functionals is again optB88-vdW, although this time, the other functionals perform almost equally well. One of the goals of the DFT investigations is to calculate the activation energy for the exfoliation of MoO_3_ nanosheets from larger MoO_3_ particles, which requires a supercell, including vacuum. As the optB88-vdW functional is known to show difficulty in electronic convergence when vacuum is present in the supercell, the DFT-D3 functional was selected as the optimal choice and will be used for all further density functional calculations in the remainder of this study.

The energies of the different molybdenum oxide structures were calculated by fully relaxing the unit cell, both the lattice parameters and atomic coordinates, using the settings and functionals given above. The energy of the paramagnetic O_2_ molecule was calculated as well, in order to compare the energies of the MoO_3_, Mo_4_O_11_, and MoO_2_ structures taking into account the change in chemical composition. To calculate the energy of the O_2_ molecule, a spin-polarized calculation was performed with the molecule at the center of a large cubic supercell of vacuum (with an edge length of 25 Å), employing the DFT-D3 functional with a *k*-mesh of 1 × 1 × 1.

The surface energy of the (010) surface of MoO_3_ was calculated by constructing a MoO_3_ supercell consisting of 5 stacked unit cells of MoO_3_ and a vacuum layer of 57 Å. This supercell was relaxed using the same settings as those used for the unit cell, but with the *k*-mesh scaled accordingly (6 × 1 × 6). The surface energy is then given by
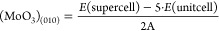
1where *A* is the area of the surface in the supercell. A factor of 2 accounts for the fact that because of the periodic boundary conditions, the supercell contains two surfaces.

Using the relaxed supercell, the activation energy required to exfoliate one layer of MoO_3_ was calculated by keeping the dimensions of the supercell fixed while shifting the upper layer upward in 10 steps, amounting to a total shift of 1.38 nm, at which point we consider the top layer to be well separated from the main slab. To evaluate the change in potential energy and a possible activation barrier associated with the exfoliation, the nudged elastic band (NEB) method of Henkelman et al.^41^ was used with a spring force constant of −5.0 eV/Å^2^.

Furthermore, to gain more insights into thermal vibrations that can cause exfoliation, AIMD simulations were performed. To this end, a 192-atom simulation cell was constructed consisting of a 2 × 3 × 2 MoO_3_ slab and a vacuum layer of more than 70 Å along the [010] axis of the stacking direction. Just as for the NEB calculation, for the AIMD simulations, the exchange and correlation energy terms were described using the PBE functional,^34^ and the DFT-D3 method^36^ was applied to account for dispersion interactions. Before the AIMD simulations were performed, the simulation cell was first fully relaxed at high accuracy. The AIMD calculations were performed at lower accuracy settings, with a cutoff energy of 400 eV for the wave functions and a cutoff energy of 560 eV for the augmentation functions, and including the Γ-point only instead of using a *k*-mesh of 3 × 1 × 3, which is customary to do when performing AIMD because of the extremely high computational cost of AIMD when the number of atoms is this large.^42,43^ With a time step of 1 fs, a 2 ps initial equilibration consisting of a ramp-up from 0 K and a 6 ps subsequent canonical (*NVT*) ensemble simulation using a Nosé thermostat^44−46^ was carried out at a simulation temperature of 300 K. The 300 K simulation was followed by a 2 ps ramp-up to 900 and 1100 K and continued at these temperatures for another 8 ps.

## Results and Discussion

### Characterization of MoO_3_ Particles

The pristine MoO_3_ particles used in this study had a broad size distribution. The particles agglomerated after being dried on the heating chip. Figure 2a,b shows the bright-field TEM images of several particles at room temperature. Figure 2c,d shows a SADP and a high-resolution TEM (HRTEM) image of the particle. Both the DP and HRTEM recordings show that the particles are oriented along the [010]-zone axis (normal to the vdW-bonded layers). This was the commonly found orientation of the particles on the support grid. The scanning electron microscopy (SEM) images displayed in Figure S1 of the Supporting Information show that the typical morphology of the particles is rounded and elongated, where the particles in general have one larger surface lying “flat” on the support. From the HRTEM images and SADP patterns shown in Figure 2 and similar recordings, it is clear that this larger surface is the (010) surface; i.e., the vdW layers are oriented parallel to the support membrane. This morphology is in agreement with calculated surface energies of the various facets, as evaluated by Sun et al.^47^ Here, the (010) surface has the lowest surface energy, which, therefore, makes up the largest facet. It was in general not possible to image the particles in other crystallographic orientations and consequently, it was not possible to image the vdW layers edge-on. Using a regular double-tilt holder and a regular grid, we intentionally tilted one of the particles in a different zone axis to observe a particle in another orientation. Figure S3b shows a DP of one particle oriented in the [410]-zone axis after tilting the particle along the *c*-axis to about 40°, starting from the commonly observed near-[010] zone axis orientation with the corresponding DP shown in Figure S3a. The DPs and HRTEM images show that the pristine MoO_3_ particles are single crystalline at room temperature.

### In Situ TEM Investigation of MoO_3_ Particles

**Figure 3:**
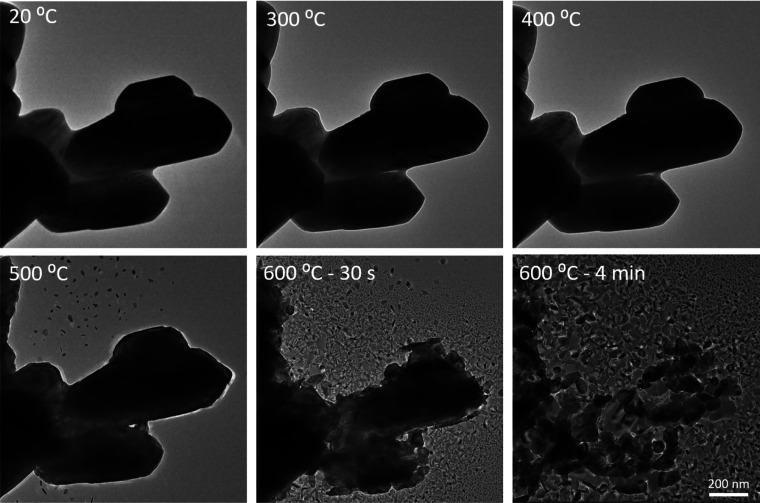
Bright-field TEM image of MoO_*x*_ particles heated from 20 °C (room temperature) to 600 °C. All images are at the same magnification.

Details of the in situ experiments are provided in the Methods section. The particles were first heated from room temperature to 600 °C in relatively large 100 °C increments. Figure 3 shows the evolution of the whole process. Below 500 °C, the morphology of particles hardly changes. At 500 °C, some small flakes appeared near the parent particles. When the temperature was raised to 600 °C, many more flakes were found around the primary particles that were formed at a rapid pace, while the larger particles broke down into smaller and smaller pieces. The disintegration of the larger particle and fast formation of the surrounding nanosheets proceeded for almost 4 min and finally, the main part of the parent particles had broken up into smaller submicrometer crystals. This process could reproducibly be imaged in multiple experiments. Videos S1 and S2 in the Supporting Information (SI) show other such events taking place when the solution is heated rapidly to 600 °C. Video S2 shows a low-resolution recording where the disintegration of some large particles can be followed simultaneously; the very thin nanosheets (giving little contrast) that are formed around the particles cannot be seen here because of the low resolution. Video S1 shows a zoomed-in recording of two particles, where the formation of nanosheets around the primary particles can be followed in real time. Here, it is observed that the surrounding nanosheets appear from one movie frame to another, indicating that the nanosheets are formed instantaneously. From these observations, we infer that the nanosheets originate from the primary particles, detaching and being expelled from the primary particles during the rapid heating process. Video S1 also shows that the nanosheets grow in the lateral size after being formed, which is possibly made possible by vapor present from partial sublimation of the MoO_3_ particles (via solid–vapor–solid growth), similar to what was previously observed during heating of WO_3_ nanoparticles.^48^ In all heating experiments, it was found that after prolonged heating at 600 °C, a number of larger particles remain that do not disintegrate further. After cooling to room temperature and subsequent examination, these larger particles were found to be completely in the MoO_2_ phase, suggesting that the formation of nanosheets stops when the larger particles have fully transformed into MoO_2_. Video S3 shows the last part of the disintegration process of the particle displayed in Figure 3.

**Figure 4:**
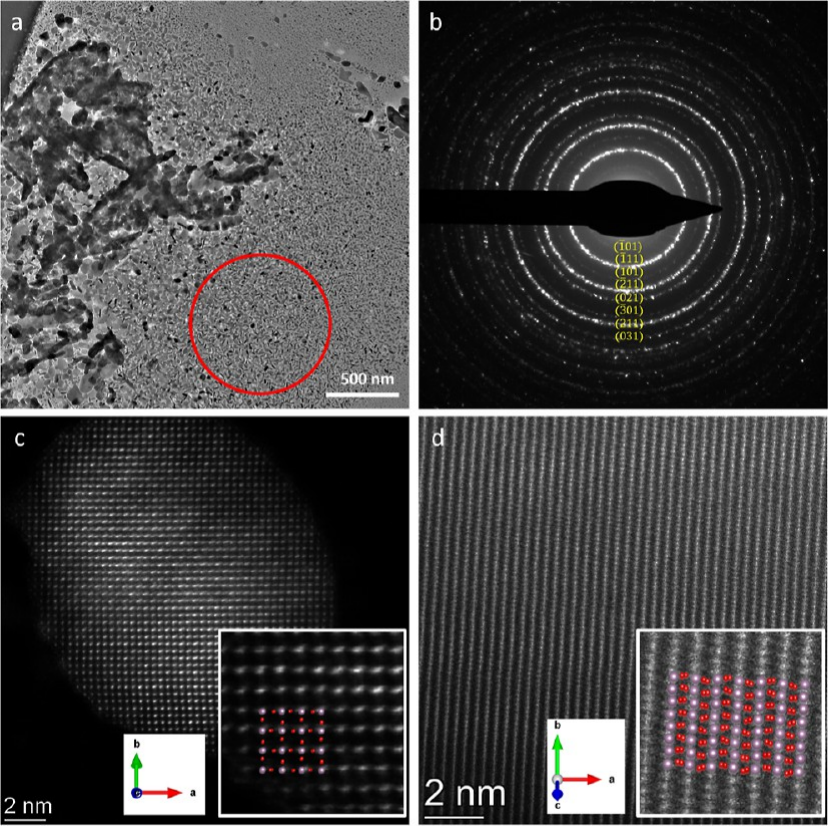
(a) Overview TEM image after heating to 600 °C and (b) diffraction pattern of the area marked in (a). The pattern was indexed, confirming the monoclinic MoO_2_ structure. (c) HAADF–STEM image of a tiny MoO_2_ particle oriented along the MoO_2_*c* zone axis (001) with in the inset a zoomed-in image of the particle with an atomic model overlay with Mo atoms (purple) and O atoms (red). (d) HAADF–STEM image of an MoO_2_ particle oriented along the MoO_2_c* zone axis (the axis perpendicular to *a* and *b* axes). The superimposed atomic model shows the positions of the Mo (purple) and the O (red) atoms.

Figure 4a shows a low-magnification overview image of the particles after being heated to 600 °C. Figure 4b shows the diffraction pattern of the area marked in (a) with a red circle. The DP was indexed and found to match that of the monoclinic MoO_2_ phase. Therefore, at 600 °C, the surrounding small flakes were reduced to MoO_2_ during the heating process. In order to analyze the nanosheets in greater detail, HAADF–STEM images were taken and are shown in panels (c) and (d), showing projections in two zone-axes (*c* and c*) of MoO_2_ at 600 °C. (The crystal structure of MoO_2_ is monoclinic, as detailed in Table 1, and c* is the axis that is geometrically orthogonal to the *a* and *b* axes.) EELS measurements were performed as well, before and after the heating, and the results are shown in Figure S4. The positions and intensities of the peaks agree with previously published results for MoO_3_ and MoO_2_,^49^ confirming the heating-induced chemical and structural transformation. We remark here that the shape of the peaks in the spectrum for MoO_2_ is slightly different from that reported in the literature data, which may be due to the fact that MoO_2_ is here present in a 2D form (which typically alters the electronic structure and therefore also the EELS spectrum). We suggest that simulation of the EELS spectra of thin layers of MoO_2_ and MoO_3_ of various thicknesses would be an interesting topic for follow-up investigations.

Figure S5 shows another group of particles at 400 and 500 °C. Figure S5 shows the zoomed-in image of the area marked with a square in (a), in which small zones in different orientations were formed in the parent particle. Figure S5 shows the diffraction pattern of the marked area in (b), which corresponds to the MoO_3_ structure. The yellow arrows mark the split spots, which is likely caused by crack formation taking place already at these lower temperatures. Figure S6 shows an SADP of a pristine particle at 500 °C in the [3̅1̅1]-zone axis. With a beam stopper, the (000) beam was blocked and more weaker peaks became visible. This confirms the formation of cracks and small domains in the larger particles before large-scale formation of MoO_2_ nanosheets, as peaks corresponding to different orientations appear.

**Figure 5:**
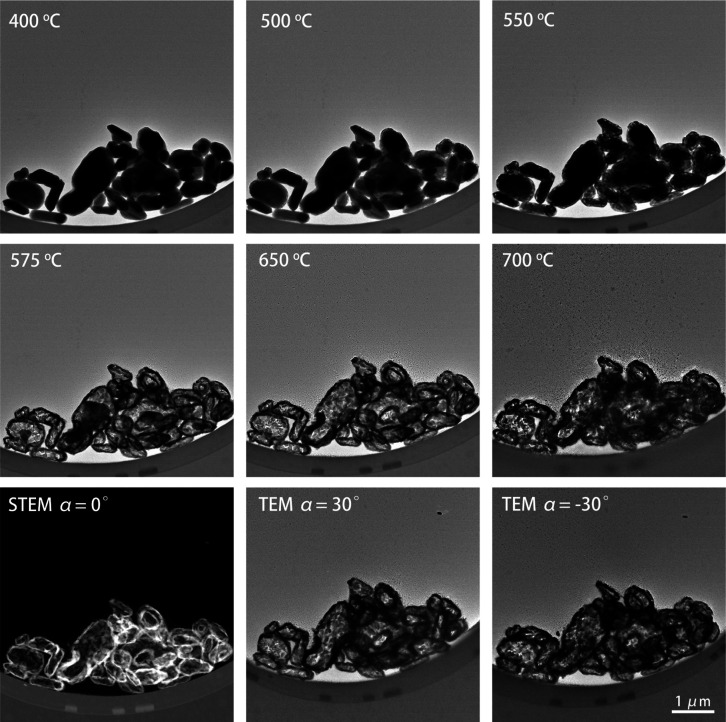
TEM images of MoO_*x*_ particles at temperatures above 400 °C. These particles were heated gently, with a 25 °C increment above 400 °C. The bottom-left image is a HAADF–STEM image obtained after heating. The last two images at the bottom-right are also recorded after heating, with the sample tilted over ±30°. Smaller MoO_2_ nanoflakes were not observed around these particles, also not when inspected at higher magnifications.

**Figure 6:**
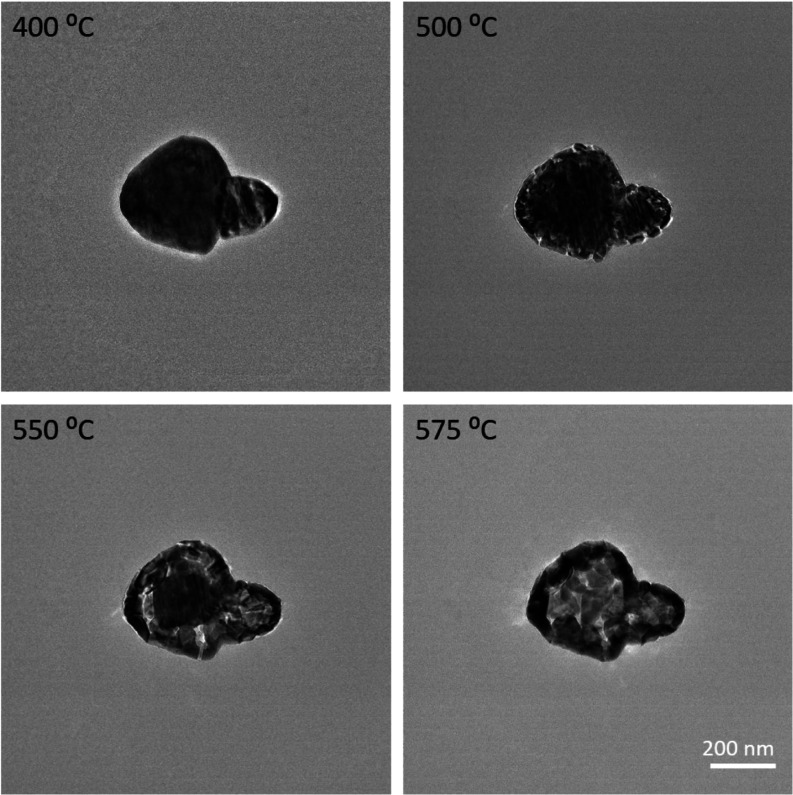
Bright-field TEM images of smaller particles heated gently with 25 °C increments to 400 °C and higher at the indicated temperatures. Smaller MoO_2_ nanoflakes were not observed around these particles, also not when inspected at higher magnifications.

As the formation of the nanosheets around the primary particles happened extremely fast in the first heating experiment, the MoO_3_ particles were also heated more gently in follow-up experiments, using smaller steps of 25 °C increments above temperatures of 400 °C. At this slower heating rate, the fast disintegration of the large particles into smaller particles did not take place, and there are much fewer flakes formed around the parent particles (shown in Figure 5). After heating to 500 °C, the edges of the particles show a brighter contrast (gray) than the central area (black) because of a different projected thickness. From 575 °C onward, however, the central areas of the particles exhibited a brighter contrast, with every particle having a dark contour, suggesting that the particles became hollow. To avoid contributions from diffraction contrast in bright-field TEM imaging (BF-TEM), a high-angle annular dark-field scanning transmission electron microscopy (HAADF–STEM) image is also recorded and is displayed at the bottom-left panel in Figure 5. In addition, BF TEM images with ±30° tilts are also taken after heating and are shown in the bottom-right panels of Figure 6. All these results show that the edges are thicker and heavier than the central area after heating and that the particles have become hollow. The corresponding DP shown in Figure S7 indicates that the hollow particles are also in the MoO_2_ phase. The thermal reduction process taking place at 500 °C was also recorded in an in situ movie, and the structural evolution in a larger particle during the process can be seen in Supporting Information, Video S4. In this movie, it is apparent that a polycrystalline configuration has formed and that the material is disappearing during the heating, making the particle thinner in projection and leaving open space behind. Although lattice fringes can be distinguished, the images do not allow one to identify a particular crystallographic direction along which the transformation progresses. Imaging at higher magnification was not possible, as then the influence of the electron beam became apparent.

To show the details of structural changes taking place during this gentle heating process, the thermal evolution of an agglomerate consisting of two smaller MoO_3_ domains is followed as well and is shown in Figure 6. At 400 °C, fringes appeared on the cluster, especially in the right domain, which is likely from bending of the lattice and a different crystallographic orientation. At 500 °C, the edges of the particles broke into small flakes, while the central area showed vertical fringes. This proceeded further inward at 550 °C and finally, the central area also broke up at 575 °C. However, the remaining particles at the center are bigger than those at the edges. This could explain what happened to the configurations shown in Figure 5. The exfoliation and cracking of the lattices started at the surface of the particle. Next, domains in different orientations formed at the surface, while the center was still single crystalline. This resulted in the bright contour at 550 °C. With increasing temperatures, small flakes also formed in the central area at a relatively slow rate. Part of the crystals sublimated during heating, while some small flakes disappeared into the vacuum of the microscope column, some of them accumulated at the edges when being expelled. Therefore, fewer flakes appeared around the parent particles, and more crystals are left at the edges of the particles.

To investigate whether the transformation into MoO_2_ already happened below 600 °C, HRTEM images of the flakes formed below 600 °C were measured and indexed, as well. Figure S8a shows an HRTEM image of a flake at 550 °C. The FFT image corresponds to an [1®3̅2]-Mo_4_O_11_ projection. The lattice spacings of MoO_3_ and Mo_4_O_11_ are very similar and in theory, the FFT image could be also indexed as [11®1] of MoO_3_. However, the (11®1) plane intersects the MoO_3_(010) vdW-bonded layers, which makes this orientation of the nanosheets extremely unlikely. Therefore, this nanosheet is most likely Mo_4_O_11_, although we cannot rule out other intermediate Magnéli phases as the corresponding interplanar placings are quite similar.^31^ As these phases were observed only as transitory phases during the dynamic in situ TEM recordings, a full crystal structure determination was not feasible. It is clear, however, that the flakes formed below 600 °C have not reduced yet to MoO_2_. Similar HR images matching the Mo_4_O_11_ phase are shown in panel (b). In at least a number of cases, the reduction from the MoO_3_ phase to the MoO_2_ phase takes place via the formation of Mo_4_O_11_ or other stoichiometrically intermediate phases.

Although it is clear from the EM images and in situ movies that nanosheets appear around the primary particles while the primary particles disintegrate into smaller and smaller subcrystals, the direct detachment of a nanoflake from a primary particle along a particular crystallographic orientation was not observed. We consider exfoliation along [010] to be most plausible; as in this direction, the layers in MoO_3_ are only bonded by weaker vdW forces, while the intralayer chemical bonds are much stronger. Unfortunately, as discussed at the beginning of the Results and Discussion section and as is also clear from the SEM images displayed in Figure S1, all particles are lying on the support grid on their largest (010) facet (with the vdW layers parallel to the support membrane) so that the (010) vdW layers could never be observed edge-on. Consequently, exfoliation from these layers could not be directly observed in the TEM. We mention here that in the literature, the (010) plane is a well-known cleavage plane of MoO_3_, which also makes it more likely that cleavage and exfoliation takes place along this particular crystallographic plane.^50,51^ As mentioned previously, the MoO_2_ nanosheets observed undergo some growth in the lateral size after being formed (Video S1), which is probably enabled by vapor that is locally present due to partial sublimation of the larger MoO_3_ particles. We expect that the observed transformations will also take place when the particles are heated ex-situ under vacuum conditions. We mention here that the precise temperature at which the transformation takes place is in general dependent on the partial oxygen pressure (thereby also being dependent on the quality of the vacuum), as was observed in our previous works on the heating-induced reduction of Co_3_O_4_ nanoparticles.^52^

One remaining question is why the massive exfoliation, as shown in Figure 3 and in Videos S1 and S3, only takes place during rapid heating. Although it is clear, also from the DFT and AIMD simulations presented below, that the weakly bound (010) layers in MoO_3_ are prone to heating-induced exfoliation, we hypothesize that the strong dependence on the heating rate is due to thermal shock taking place at the nanoparticle as the temperature of the MEMS heater is swiftly increased. The fast heating rate results in an out-of-equilibrium process involving shockwise heat transport, likely leading to high shear stresses between the weakly bound layers and resulting in efficient cleavage and exfoliation and scattering of exfoliated flakes around the primary particles. We mention here that in the literature, so-called liquid phase exfoliation (LPE) by ultrasonication is known as a standard method of delamination and exfoliation of vdW-bonded materials,^8,53^ and we hypothesize that a similar process is taking place in the experiments during rapid heating.

### DFT Calculations

To gain more insights into the relative stability and energetics of the observed phases, DFT calculations were performed. The MoO_3_, Mo_4_O_11_, and MoO_2_ structures were first relaxed using the DFT-D3 functional, as described in the Methods section. Also the energy of the O_2_ molecule was calculated. The optimized O_2_ bond length is 1.23 Å, which agrees well with a reference value of 1.21 Å.

The reduction reaction of MoO_3_ to MoO_2_ is given by
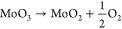
1aand from the total energies of these three phases, it follows that there is an energy cost of 1.97 eV per MoO_3_ formula unit (f.u.) that is reduced to MoO_2_. In a similar way, the reduction reaction of MoO_3_ to Mo_4_O_11_ is given by
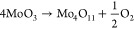
2the energy cost of which is 0.31 eV/f.u. of MoO_3_. The DFT calculations yield formation enthalpies valid for a temperature of 0 K and a pressure of 0 Pa. Because the heating was performed under high vacuum, we assume that the oxygen pressure is zero and that entropy can be neglected. Because there is an energy cost associated with the reduction of MoO_3_, the reduction is not energetically favorable at 0 K. Because the energy cost to form Mo_4_O_11_ is lower than the cost to form MoO_2_, it is expected that Mo_4_O_11_ will form before MoO_2_. It is also possible to form Mo_4_O_11_ in a reaction between MoO_3_ and the already formed MoO_2_
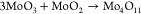
3which actually results in an energy gain of 0.25 eV/f.u. of MoO_3_. From the DFT calculations, it cannot be inferred whether the found Mo_4_O_11_ is formed as an intermediate or in a side reaction. There was little evidence of Mo_4_O_11_ in the larger particles and only at 500 and 550 °C. It is, therefore, possible that Mo_4_O_11_ is only an intermediate phase that quickly reduces further to MoO_2_. Another possibility is that Mo_4_O_11_ is formed in a side reaction, after which it also reduces to MoO_2_ at higher temperatures. Because the side reaction is energetically favorable at 0 K, it is more likely that Mo_4_O_11_ will form in the side reaction.

In order to calculate the (010) surface energy, an MoO_3_ supercell consisting of a 5-layer slab was also relaxed, using the same settings and functional as for the MoO_3_ unit cell. The surface energy of the (010) MoO_3_ surface is calculated by using eq 1. With a surface area of 14.46 Å^2^ as calculated from the lattice parameters of the relaxed MoO_3_ supercell, the surface energy was calculated to be 1.17×10^–2^ eV/Å^2^, or 0.187 J m^–2^ using eq 1.

**Figure 7:**
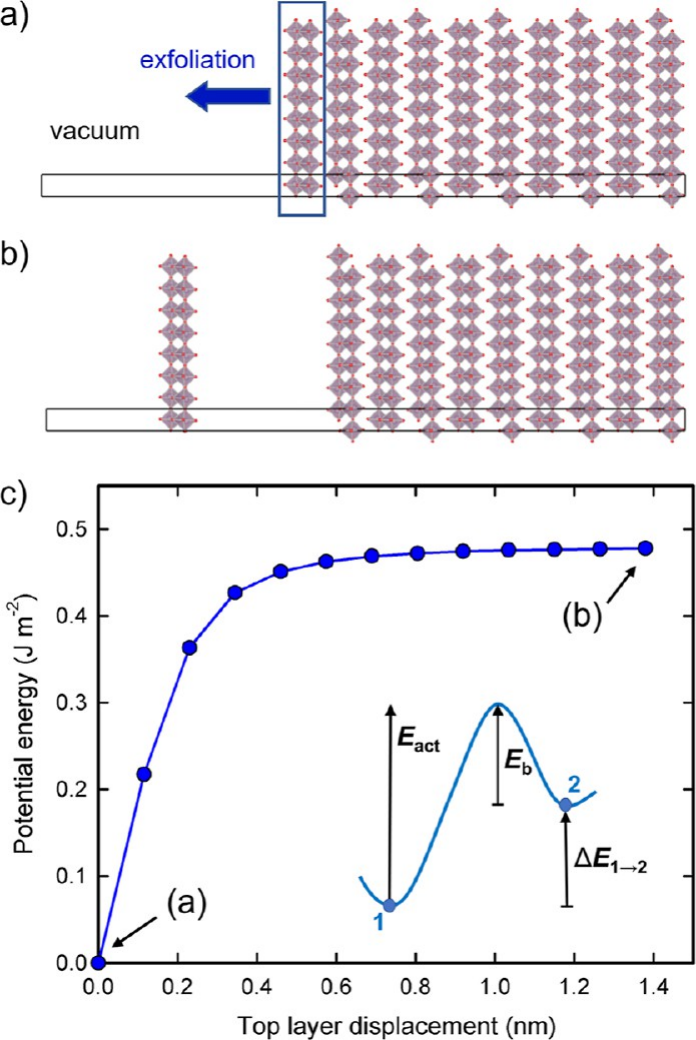
(a,b) Supercell that was used to calculate the activation energy for exfoliation, with (a) MoO_3_ supercell before exfoliation and (b) largest calculated shift of the top layer of 1.38 nm. Black lines indicate the boundaries of the supercell which includes a substantial vacuum layer. The periodic cell is repeated in lateral dimensions several times to display the slab-like nature of the supercell. The color code for the atoms is the same as the code in Figure 1. (c) Evolution of the potential energy of MoO_3_ as a function of the shift of the exfoliated top layer calculated by using the NEB method. The inset shows a schematic potential energy diagram for a transition from point 1 to point 2 with the activation barrier *E*_b_, the difference in potential energy Δ*E*, and the activation energy *E*_act_.

The activation energy required for the exfoliation will be at least twice the surface energy (as otherwise exfoliation would take place spontaneously) and is higher when an additional energy barrier needs to be overcome. To calculate the activation energy for exfoliation of the (010) MoO_3_ layers as observed during heating in the TEM, the upper (010) layer was shifted away from a thicker MoO_3_ slab, and the potential energy of the supercell was calculated along that pathway using the NEB method, as described in the Methods section. Figure 7a shows the configuration of the slab before shifting of the top layer, Figure 7b shows the configuration with a fully exfoliated top layer, and Figure 7c shows the potential energy evolution during the shift. It is known from the transition-state theory that an energy barrier often needs to be overcome when going from one minimum or platform in potential energy to another minimum or platform in potential energy.^54,55^ The inset in panel (c) shows schematically the activation energy for the general case of an unfavorable transition (Δ*E*_pot_ > 0) with energy barrier *E*_b_. This activation energy can be supplied by, e.g., thermal (kinetic) energy. When comparing the schematic potential energy diagram with the calculated potential energy curve in panel (c), it is clear that there is no energy barrier associated with exfoliation. In other words, the activation energy is equal to the total difference in the potential energy. The likely reason for the absence of an energy barrier is that the vdW interactions between the (010) layers are mainly ruled by electric dipole–dipole interactions, which become weaker with increasing distance, so that no barrier is to be expected. In Figure 3c, the energy cost to remove one layer from the MoO_3_ bulk is found to be 0.478 J m^–2^. This energy is higher than the energy required to create two (010) surfaces [2× the (010) surface energy of 0.187 J m^–2^ equals 0.374 J m^–2^]. The absence of an energy barrier indicates that the MoO_3_ layers are weakly bound.

### Ab Initio Molecular Dynamics Simulations

In general, chemical reactions cannot be simulated by means of DFT because the formalism is only valid for the electronic ground state, due to the second Hohenberg–Kohn theorem that relies on the variational principle.^56^ However, the (010) layers in MoO_3_ are only bonded by vdW attractions (mainly electric dipole–dipole interactions) and are, therefore, more physical in nature, in contrast to the breaking of chemical bonds (typically characterized by the formation of new molecular orbitals or transfer of electrons). Because of the physical vdW bonding, the configuration can be considered to be still very close to the electronic ground state and therefore, the use of DFT to simulate the interlayer interactions is reasonably justified. Having said that, with increasing temperature, the simulated system will move further away from the electronic ground states, e.g., electronic excitations are not incorporated in the DFT calculations. Nonetheless, because of its foundation on quantum mechanics, AIMD is considered to be quite reliable for simulations at elevated temperature in comparison to, for example, force-field molecular dynamics simulations.

**Figure 8:**
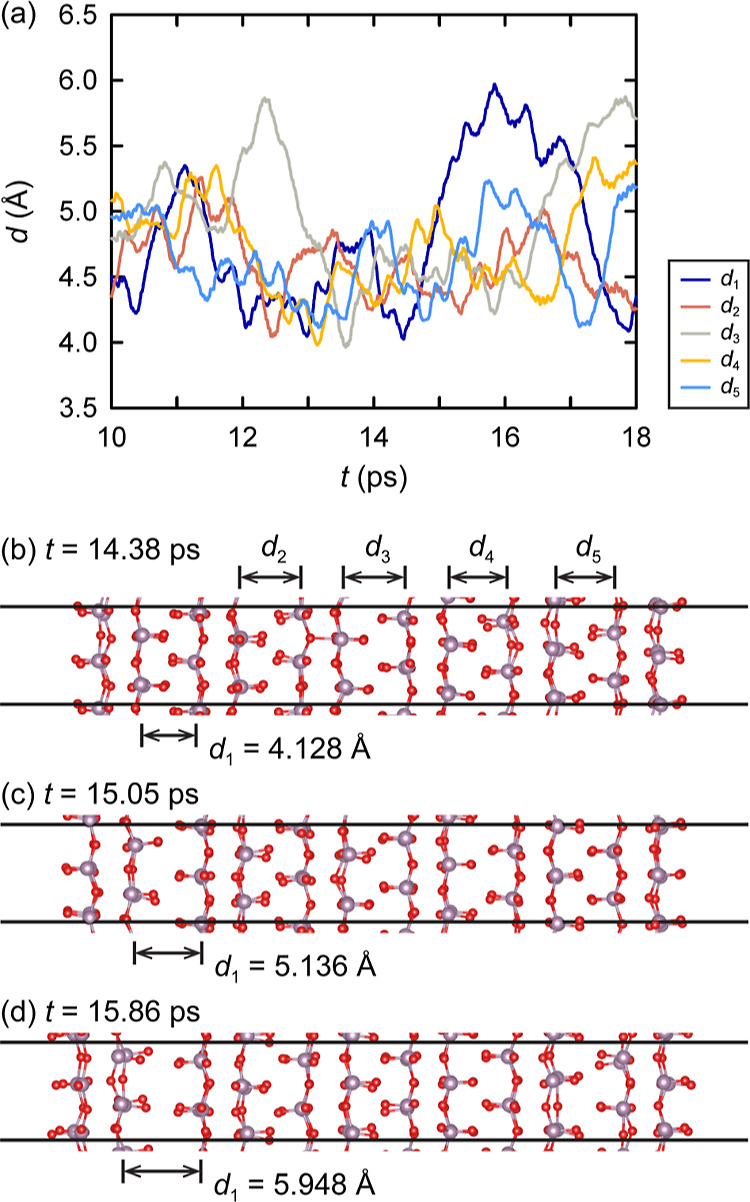
AIMD simulation of the 2 × 3 × 2 MoO_3_ slab at 900 K. (a) Separating distances, as observed during the simulation. The definition of the distances is illustrated in panel (b). (b–d) Snapshots of three configurations leading up to the configuration in which the maximum separation occurred, showing on their left-hand side the onset of exfoliation of the outermost layer from the slab. Here, the Mo and the O atoms are represented by mauve and red spheres, respectively. The boundaries of the simulation cell are indicated by black solid lines.

To obtain more insights into the thermal vibrations leading to exfoliation, AIMD simulations were performed using a 192-atom simulation cell consisting of a 2 × 3 × 2 MoO_3_ slab and a vacuum layer of more than 70 Å along the [010] axis of the stacking direction, as described in the Methods section. In Figure 8 and in Figure S9 in the Supporting Information, the temperature and the separation between Mo layers are shown as observed during the AIMD simulations. Here, the separation between Mo layers is defined as the averaged perpendicular distance between the two closest Mo atomic layers of two adjacent MoO_3_ bilayers (the definition of the individual separating distances *d*_1_, *d*_2_, *d*_3_, *d*_4_, and *d*_5_ can be found illustrated in Figure S9c). As can be seen, the separation between Mo layers, which was calculated to be ∼4.17 Å at 0 K, increases at elevated temperatures.

It should be noted that for the AIMD simulations, the dimensions of the supercell are fixed. This means that during the simulations thermal expansion of the system can only be accommodated in the stacking direction of the MoO_3_ layers. At the same time, MoO_3_ is known to exhibit anisotropic thermal expansion, where with increasing temperature the lattice expands considerably along the stacking direction but only a little along the [100] direction, and even slightly contracts along the [001] direction.^57^ To get a general idea of the effect of the fixed cell parameters and the limited time and length scale of the AIMD simulations on the observed separating distances, we compared the increase of the separation in the middle (*d*_3_), which is the most bulk-like part of the slab, as obtained during the 300 K simulation with previous experimental results on the thermal lattice expansion of MoO_3_ bulk.^57^ The average value of *d*_3_ during the 300 K simulation was found to be 4.23 Å, which is an increase along the stacking direction of 1.4% relative to the 0 K value. Experimentally, for MoO_3_ bulk, a lattice expansion of 0.9% has been indicated for this temperature range along the stacking direction, and expansions of 0.2 and −0.04% along the other two directions.^57^ This indicates that the effect of the fixed cell parameters with only the possibility of expansion along the stacking direction and the limited time and length scale of the simulations on the observed values of *d* can be considered to be small.

The maximum separation observed during the 300 K simulation has a value of 4.88 Å, which is an increase of ∼0.7 Å relative to the 0 K value. A snapshot of the configuration in which this maximum separation occurred can be found in Figure S9d and shows that the simulation at 300 K, which is in agreement with our experimental results, does not point to the possibility of exfoliation at this temperature.

The picture is quite different for the simulations at 900 and 1100 K. The maximum separation observed during the 900 K simulation has a value of 5.97 Å (see Figure 8a) and involves the moving away of an outermost layer (see Figure 8b–d and Video S5). This movement away clearly qualifies as the onset of exfoliation of this layer from the slab. Not only did its separating distance increase by ∼1.8 Å relative to the value at 0 K; as can be seen from the configurations depicted in Figure 8b–d (and see also Figure S9i and Video S5), the moving away of this layer along the stacking direction was accompanied by a shifting of the layer along the [001] direction by which the oxygen atoms alongside the formed gap started occupying positions more opposite of each other (but only in this direction). A similar movement and shifting away was observed for the layers involved in the maximum separation occurring during the 1100 K simulation. In this case, the gap formed in the middle of the slab (see Figure S9l–n), which suggests the onset of separation of the slab into two-halves rather than the onset of the exfoliation of a single layer from the slab, and resulted in a maximum separating distance of 6.13 Å (see Figure S9k), which is an increase of ∼2.0 Å relative to the value at 0 K. Such an onset of separation of the slab into two-halves was observed during the 900 K simulation as well (see Video S5), with a maximum value of *d*_3_ of 5.88 Å (see Figure 8a), which is an increase of ∼1.7 Å relative to the value at 0 K. The averaged value of *d*_3_ during the 900 K simulation was found to be 4.95 Å and corresponds to an increase of 18.6% relative to the 0 K value, while in the mentioned previous experimental study on MoO_3_ bulk a lattice expansion of 4.2% has been reported for the 0–900 K energy range.^57^ This is yet another indication that at temperatures of 900 K and higher, exfoliation and separation of single- and multilayer MoO_3_ nanosheets will very likely take place.

At the limited time scale of the AIMD simulations presented here, full delamination was not observed. Structural features, such as terrace steps at the (010) surface and edges, which are expected to serve as starting points for delamination during our experiments, were not incorporated into the simulations. Considering the quite extreme additional separation between the layers, of up to ∼1.8 Å for the 900 K simulation, full delamination events can very well expected to occur, however, for prolonged simulation times.

## Conclusions

In this study, the thermal evolution of micrometer-sized MoO_3_ particles was investigated. When heated rapidly, the large, monocrystalline particles broke up into smaller crystals starting at around 500 °C. The disintegration of the MoO_3_ particles started from the surface and progressed toward the center of the particles. Upon rapid annealing at a higher temperature of 600 °C, the thermal reduction of the MoO_3_ particles took place at a very high pace while very thin MoO_2_ nanosheets were formed on the substrate in a wide area around the primary particles. SADP recordings and high-resolution STEM images show that the nanosheets have the MoO_2_ crystal structure and are lying on the substrate in a (001) orientation. The nanosheets undergo slight lateral growth after their formation, which is likely due to vapor present from partial sublimation of the larger MoO_3_ particles with growth taking place via solid–vapor–solid growth. The formation of MoO_2_(001) nanosheets was observed only with rapid heating.

Upon gentle annealing, MoO_3_ particles were reduced to MoO_2_ without high-pace disintegration. Here, the initial morphology was more or less retained, but in this case, the initially single-crystal MoO_3_ micron-sized particles turned into hollow structures having a polycrystalline MoO_2_ shell.

DFT calculations were performed to obtain insights into the energetics of the transformation. Taking into account vdW interactions was found to give a major improvement in the prediction of the *b* lattice parameter of bulk MoO_3_, which is along the layer stacking direction. Using the DFT-D3 functional, the surface energy of the MoO_3_(010) surface was calculated to be 0.187 J m^–2^ and the activation energy for exfoliation of a single MoO_3_ layer was calculated to be 0.478 J m^–2^. The energy path showed that the activation energy for the exfoliation is equal to the potential energy change before and after the exfoliation, meaning that the layers in MoO_3_ are weakly bound and that there is no additional activation barrier. AIMD simulations were also performed and showed that thermal vibrations result in strong fluctuations of the (010) interlayer distances, where for a temperature of 900 K the additional separation between the layers can be as large as 1.8 Å (in comparison to the separation at 0 K), marking the onset of exfoliation. Both DFT and AIMD simulations show that the (010) layers are weakly bound and prone to delamination. The reason that the exfoliation takes place experimentally only for high heating rates is most likely due to thermal shock resulting in mechanical stresses inside the primary particles, which not only causes delamination but also scattering of the exfoliated flakes over considerable distances away from the primary particles.

Our study shows that depending on the heating rate, micron-sized MoO_3_ particles can either be turned into hollow structures with polycrystalline shells, or can be nearly completely disintegrated into thin MoO_2_(001) nanosheets when heated at a very high pace at a temperature of 600 °C. The efficient production of molybdenum oxide nanosheets with a very large effective surface area is interesting for, e.g. applications in catalysis.

## Supporting Information Available

SEM images, typical heating profiles, TEM, ED, and EELS data, AIMD simulation results, and lattice reflections of MoO_3_, MoO_2_, and Mo_4_O_11_ (PDF)
In situ TEM recordings and AIMD simulations (MP4)
Low-resolution recording of the disintegration of some large particles (MP4)
Video of the last part of the disintegration process of the particle (AVI)
Video of structural evolution in a larger particle during the thermal reduction process (MP4)
Video of separation of the slab into two-halves (AVI)

## Supplementary Material

jp3c05159_si_003.mp4

jp3c05159_si_004.avi

jp3c05159_si_005.mp4

jp3c05159_si_006.avi
