# Supplementary material for: Thermal Reduction of MoO3 Particles and Formation of MoO2 Nanosheets Monitored by In Situ Transmission Electron Microscopy
Source: J Phys Chem C Nanomater Interfaces. 2023 Oct 26;127(43):21387–98. doi: 10.1021/acs.jpcc.3c05159 (PMC10626599; doi:10.1021/acs.jpcc.3c05159)
Supplement: Supplementary file 1 — jp3c05159_si_001.pdf [file jp3c05159_si_001.pdf]

Supporting Information

Thermal Reduction of MoO_3 Particles and Formation of MoO_2 Nanosheets Monitored by In-Situ Transmission Electron Microscopy

Xiaodan Chen[1], Roos M. de Boer[1], Ali Kosari[1,2], Heleen van Gog[3], Marijn A. van Huis[1,2]

[1] *Soft Condensed Matter, Debye Institute for Nanomaterials Science, Utrecht University, Princetonplein 5, 3584 CC Utrecht, The Netherlands*

[2] *Electron Microscopy Centre, Utrecht University, Universiteitsweg 99, 3584 CG Utrecht, The Netherlands*

[3] *Nanostructured Materials and Interfaces, Zernike Institute for Advanced Materials, University of Groningen, Nijenborgh 4, 9747 AG Groningen, The Netherlands*

Supporting Figures

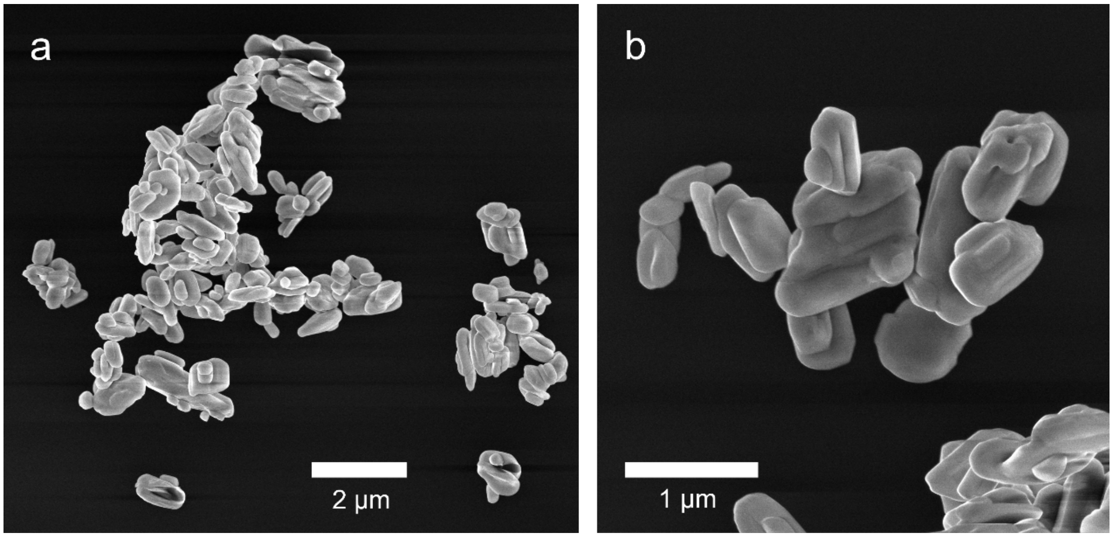

Figure S1. Scanning electron microscopy (SEM) images of the as-received MoO_3 particles dropcast onto a regular TEM grid.

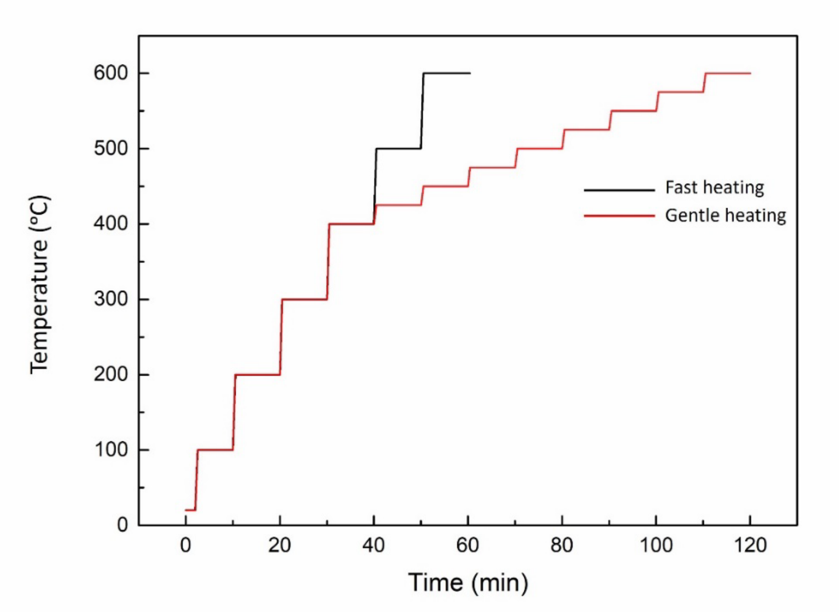

Figure S2. Typical heating temperature profiles used in the experiments. In the case of gentle heating (red curve) the temperature was increased with increments of 25 °C between 400 and 600 °C, in the case of rapid heating (black cuve) the temperature was increased with increments of 100 °C between 400 and 600 °C. The experiments were repeated several times. The typical holding times were 10 mins; during some experiments the temperature was kept for longer durations at a particular step to allow more extensive inspections, which was found not to affect the intermediate or final results.

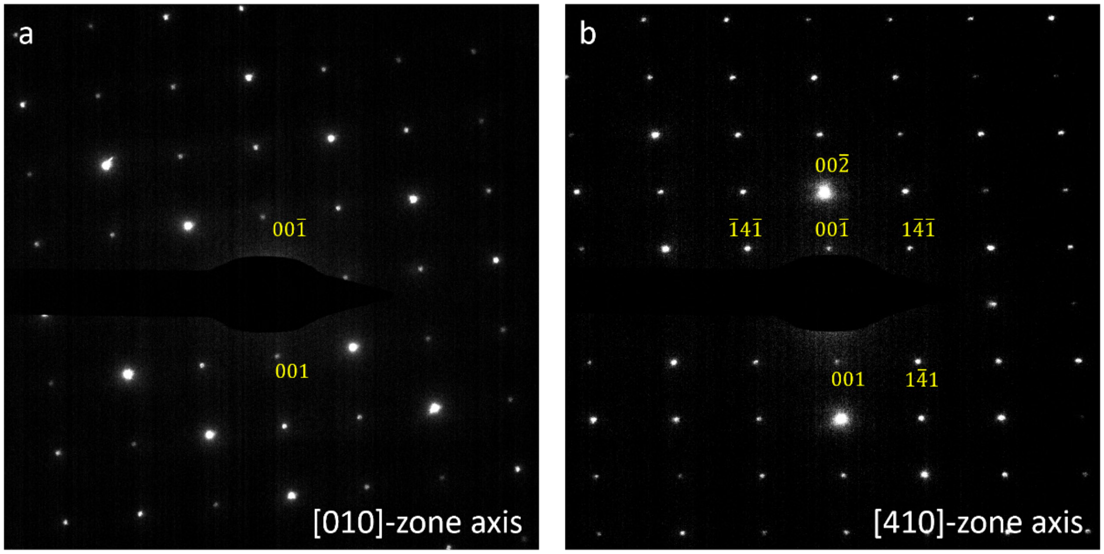

Figure S3. Selected area diffraction patterns (SADPs) of a pristine MoO_3 particle recorded at room temperature. (a) Near [010] zone axis orientation. (b) [410]-zone axis orientation. (b) was taken after tilting the particle along the c-axis to about 40 degrees.

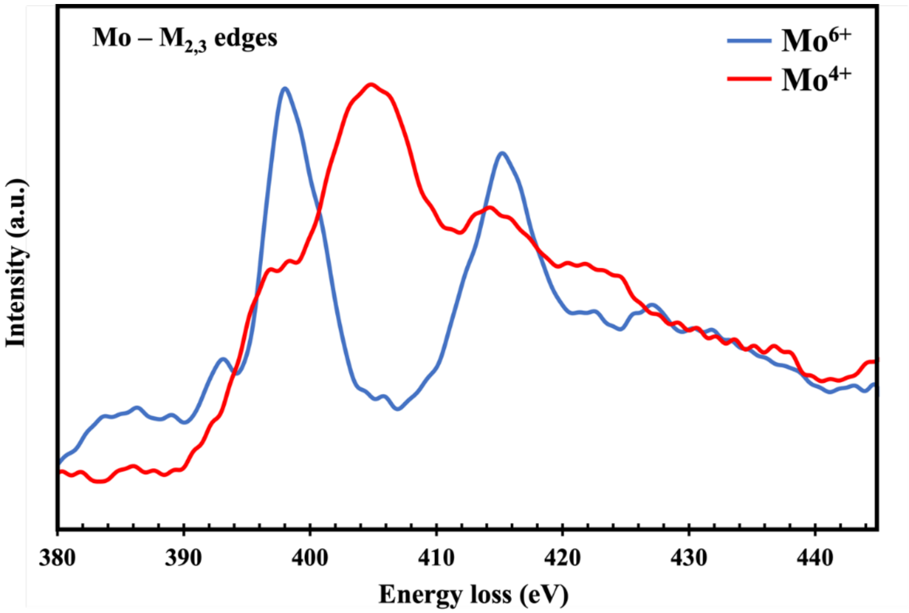

Figure S4. EELS spectra of MoO_3 and MoO_2 showing the Mo-M2,3 edges. The background continuum was removed before the onset of the Mo-M2,3 edge using a power law model. The effect of thickness was minimized by removing the plural scattering event with the log-ratio method using the zero-loss peak of the corresponding spectra, although the spectra were collected from thinnest regions in the specimens in STEM mode.

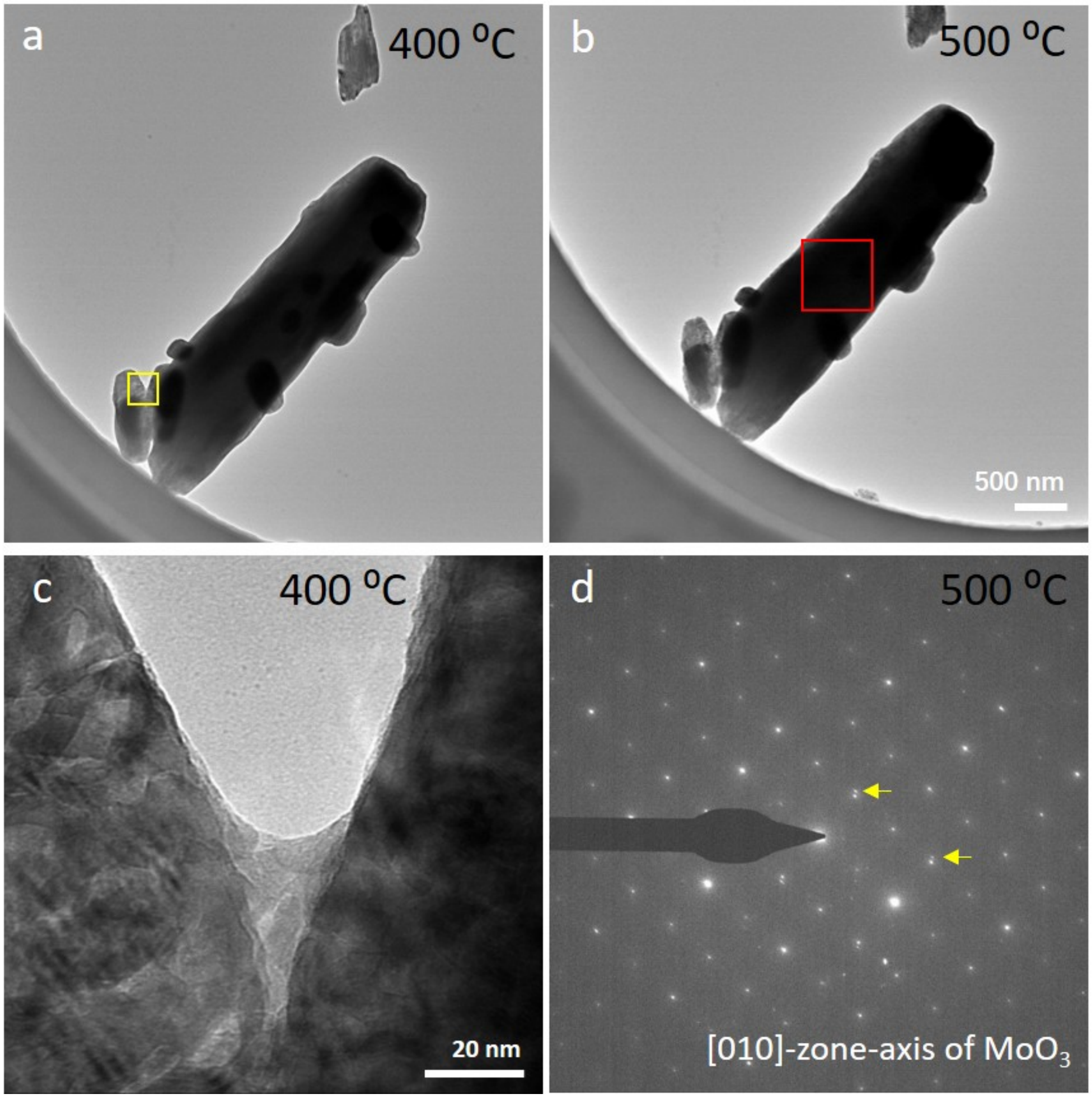

Figure S5. (a,b) BF-TEM images at 400 °C and 500 °C, respectively, recorded just after the respective heating steps. (c) TEM image of the marked area in (a), showing the details. (d) SADP of the marked area in (b), indicating the MoO_3 structure observed in [010]-zone axis. Some of the spots in (d) split (marked by yellow arrows), indicating crack formation where subcrystals are in a small tilt angle with respect to each other.

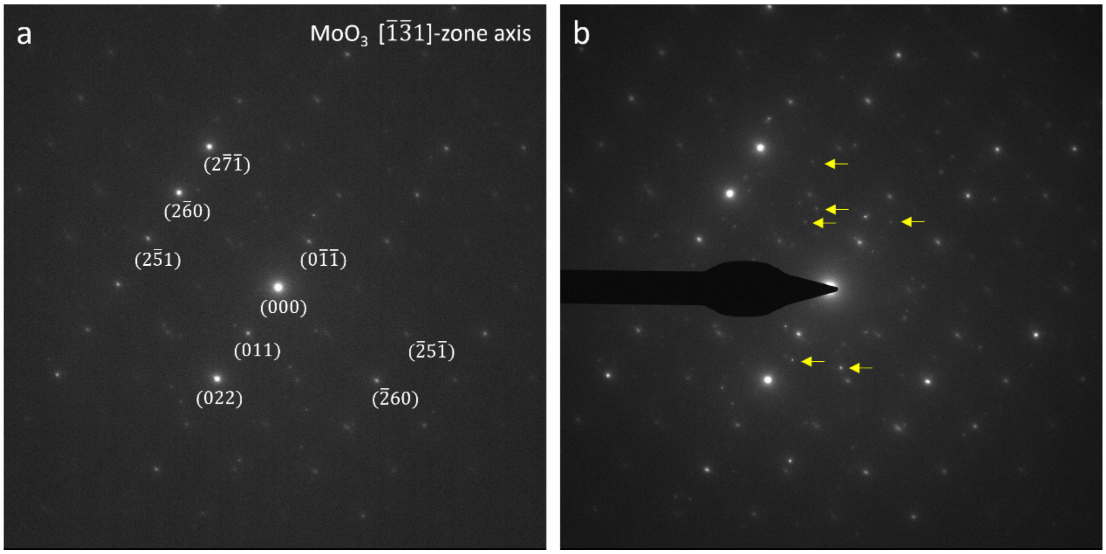

Figure S6. SADPs at 500 °C with MoO_3 in the [$\bar{1}\bar{3}1$]-zone axis. (a) without beam stopper; (b) with beam stopper. Yellow arrows mark the new peaks that appeared when the beam stopper was used. These peaks corresponds to different orientations, confirming the formation of cracks and small domains before the breaking up of the particle.

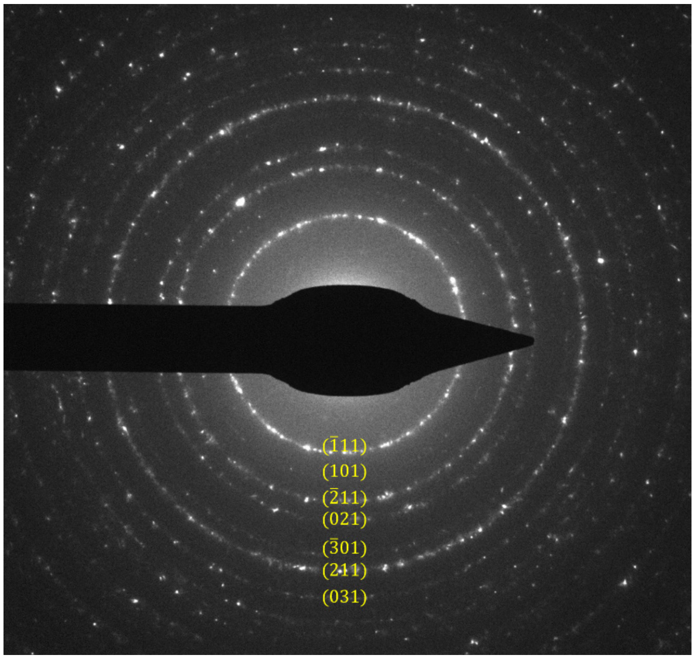

Figure S7. SADP of the particles after gentle heating to 600 °C, showing the MoO_2 structure.

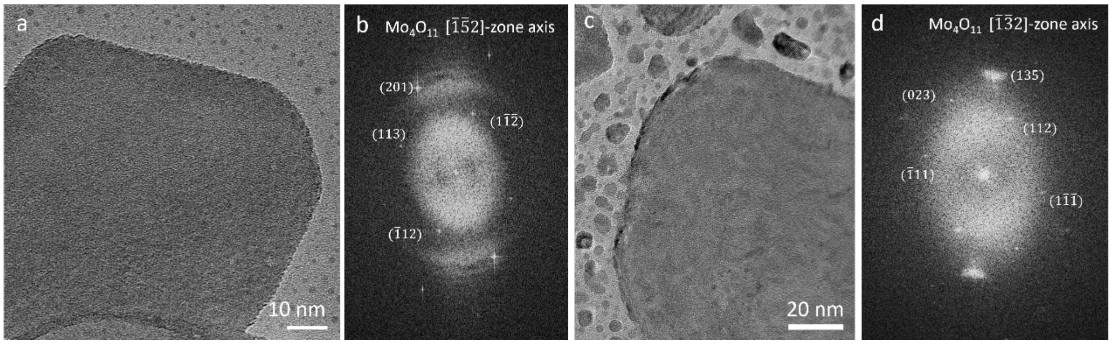

Figure S8. HRTEM images (a,c) and corresponding FFTs (b,d) of larger flakes that were formed at temperatures of (a,b) 550 °C and (c,d) 500 °C. Some remaining thermal drift in the heating holder leads to a cut-off of the higher spatial frequencies in horizontal direction which is visible in the FFTs.

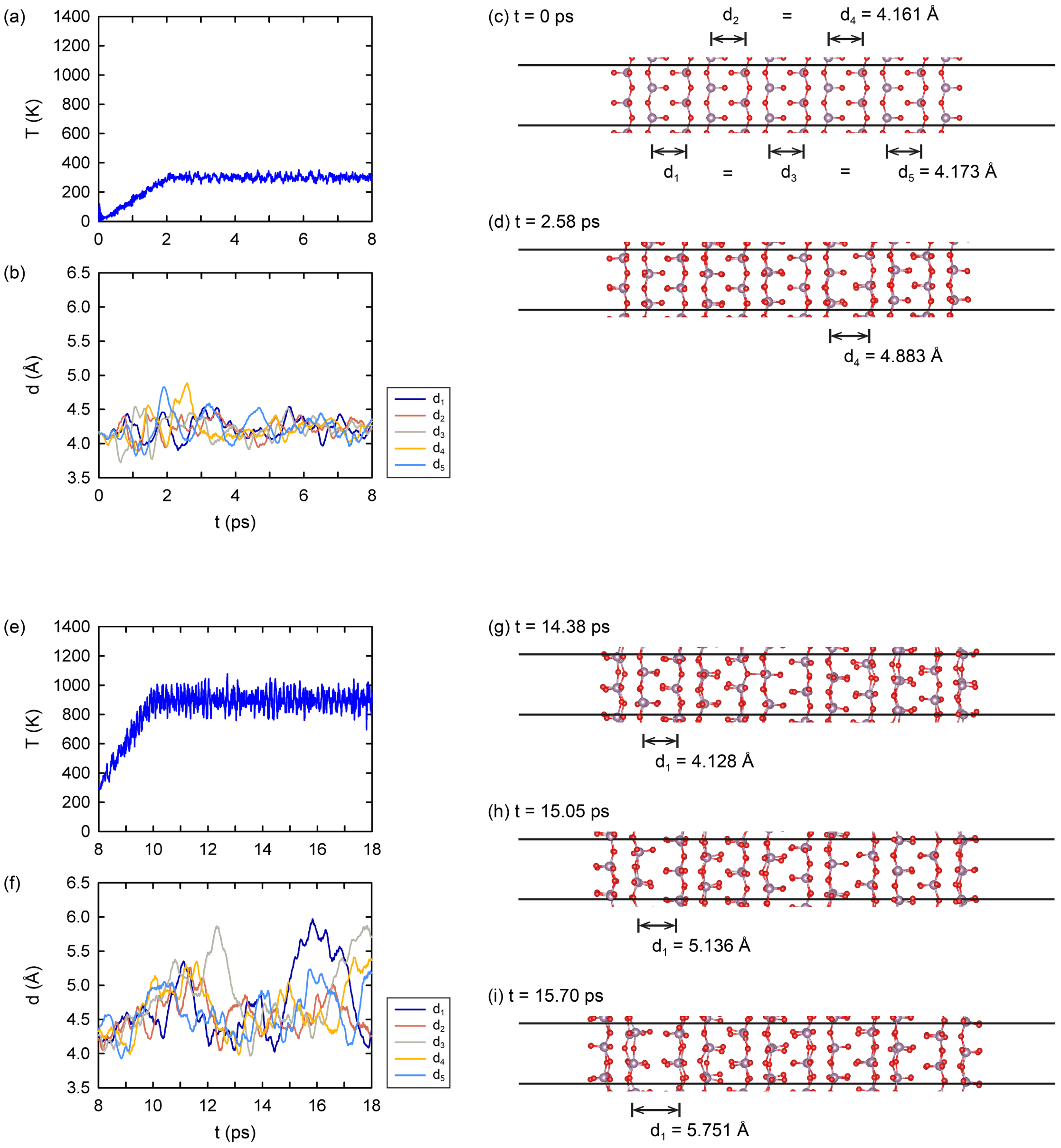

Figure S9. AIMD simulation of the 2×3×2 MoO_3 slab. Panels (a,b), (e,f), and (j,k) (see next page) show the temperature and the separating distances as observed during the simulations. The definition of the distances is illustrated in panel (c) showing the starting configuration corresponding to temperature of 0 K. Panel (d) shows a snapshot of the configuration in which the maximum observed separation during the 300-K simulation occurred. Panels (g–i) and (l–n) (see next page) show snapshots of three configurations leading up to the configuration in which at 900 and 1100 K the maximum separation occurred. Mauve and red spheres denote Mo and O atoms, respectively. The boundaries of the simulation cell are indicated by black solid lines.

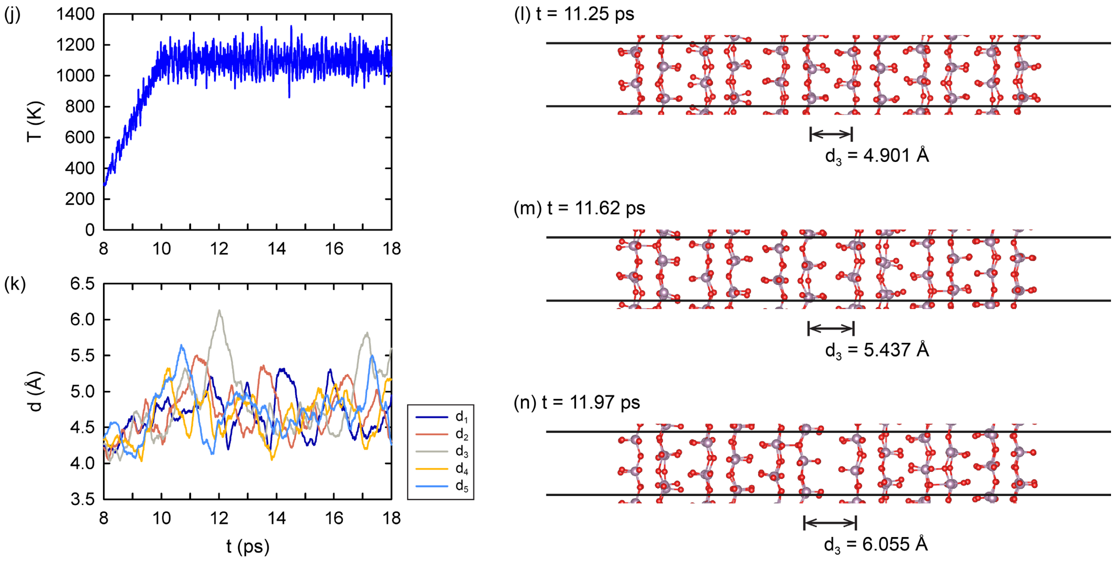

Figure S9, continued. For caption, see previous page.

Supporting Tables

Table S1. Lattice reflections of MoO_3 (JCPDS 05-0506)

No.	h	k	l	d [A]	2Theta[deg]	I [%]
1	0	2	0	6.98000	12.672	15.0
2	1	1	0	3.82000	23.267	85.0
3	0	4	0	3.47000	25.652	20.0
4	1	2	0	3.44000	25.879	40.0
5	0	2	1	3.27000	27.250	100.0
6	1	3	0	3.01000	29.655	10.0
7	1	0	1	2.70700	33.065	40.0
8	1	1	1	2.65700	33.706	70.0
9	1	4	0	2.61300	34.291	5.0
10	0	4	1	2.52800	35.481	15.0
11	1	3	1	2.33700	38.490	30.0
12	0	6	0	2.31200	38.923	20.0
13	1	5	0	2.27700	39.546	25.0
14	1	4	1	2.13500	42.298	25.0
15	2	0	0	1.98200	45.741	40.0
16	2	1	0	1.96500	46.159	50.0
17	2	2	0	1.90700	47.649	5.0
18	0	0	2	1.85200	49.156	80.0
19	2	3	0	1.82200	50.020	35.0
20	0	2	2	1.78800	51.039	20.0
21	1	6	1	1.75400	52.102	5.0
22	0	8	0	1.73500	52.716	65.0
23	2	4	0	1.72000	53.212	5.0
24	2	2	1	1.69400	54.094	40.0
25	1	1	2	1.66600	55.080	70.0
26	1	2	2	1.63000	56.403	40.0
27	1	7	1	1.59900	57.598	20.0
28	1	3	2	1.57700	58.479	10.0
29	0	8	1	1.56900	58.806	15.0
30	2	6	0	1.50600	61.526	5.0
31	2	5	1	1.47900	62.775	30.0
32	0	6	2	1.44600	64.378	20.0
33	1	5	2	1.43500	64.931	25.0
34	2	7	0	1.40200	66.656	10.0
35	2	0	2	1.35300	69.407	35.0

36	2	1	2	1.34700	69.761	40.0
37	2	2	2	1.32800	70.907	5.0
38	0	10	1	1.30000	72.675	45.0
39	2	4	2	1.26000	75.374	5.0

Table S2. Lattice reflections of MoO_2 (JCPDS 32-0671)

No.	h	k	l	d [A]	2Theta[deg]	I [%]
1	-1	0	1	4.80500	18.450	2.0
2	-1	1	1	3.42000	26.033	100.0
3	1	0	1	2.81300	31.785	4.0
4	2	0	0	2.44200	36.775	30.0
5	1	1	1	2.43700	36.853	30.0
6	-2	1	1	2.42600	37.026	70.0
7	-2	0	2	2.40300	37.393	35.0
8	2	1	0	2.18100	41.365	6.0
9	0	2	1	2.17100	41.564	2.0
10	-2	1	2	2.15600	41.867	5.0
11	-3	0	1	1.84100	49.469	11.0
12	2	1	1	1.72500	53.045	30.0
13	-2	2	0	1.72300	53.112	35.0
14	-3	1	2	1.71100	53.514	40.0
15	-2	2	2	1.70900	53.581	35.0
16	-2	1	3	1.69760	53.970	20.0
17	-3	0	3	1.60330	57.429	1.0
18	-3	1	0	1.54430	59.842	7.0
19	0	3	1	1.53600	60.198	13.0
20	0	1	3	1.52720	60.581	9.0
21	-3	2	1	1.46760	63.319	4.0
22	2	0	2	1.40570	66.458	4.0
23	-4	0	2	1.40190	66.661	20.0
24	-2	0	4	1.38450	67.611	5.0
25	3	0	1	1.35480	69.301	2.0
26	0	3	2	1.34480	69.891	1.0
27	-3	2	3	1.33810	70.293	1.0
28	-4	1	1	1.30330	72.461	5.0
29	-4	1	3	1.29120	73.250	5.0
30	-3	1	4	1.28290	73.802	1.0
31	4	0	0	1.22190	78.161	7.0
32	2	3	1	1.21750	78.498	10.0
33	1	3	2	1.21460	78.721	6.0

34	-2	3	3	1.20760	79.267	7.0
35	-2	2	4	1.20280	79.647	4.0
36	3	2	1	1.18370	81.197	2.0
37	1	2	3	1.17640	81.808	1.0
38	-3	3	0	1.14850	84.242	2.0
39	0	3	3	1.14140	84.889	4.0

Table S3. Lattice reflections of Mo_4O_{11} (JCPDS 05-0337)

No.	h	k	l	d [A]	2Theta[deg]	I [%]
1	2	0	0	12.30000	7.181	30.0
2	3	0	1	5.19000	17.071	10.0
3	4	0	1	4.53000	19.581	25.0
4	0	1	1	4.24000	20.935	45.0
5	2	1	1	4.00000	22.206	100.0
6	5	0	1	3.95000	22.491	65.0
7	3	1	1	3.75000	23.707	95.0
8	6	0	1	3.48000	25.577	85.0
9	1	0	2	3.33000	26.750	10.0
10	6	1	0	3.26000	27.335	20.0
11	3	0	2	3.11000	28.681	15.0
12	2	1	2	2.78700	32.090	50.0
13	5	0	2	2.77100	32.280	15.0
14	0	2	0	2.72200	32.878	60.0
15	3	1	2	2.70000	33.153	45.0
16	8	1	0	2.66300	33.627	70.0
17	6	0	2	2.59300	34.563	30.0
18	2	2	1	2.47300	36.297	10.0
19	7	0	2	2.42300	37.073	12.0
20	10	1	0	2.22500	40.510	30.0
21	2	0	3	2.20700	40.855	15.0
22	6	2	1	2.14600	42.071	35.0
23	7	2	1	2.04600	44.233	10.0
24	10	0	2	1.97500	45.912	30.0
25	5	2	2	1.94100	46.764	45.0
26	6	2	2	1.87700	48.459	40.0
27	11	0	2	1.85200	49.156	35.0
28	7	2	2	1.81000	50.375	30.0
29	7	1	3	1.78400	51.161	35.0
30	1	3	1	1.75000	52.230	10.0
31	2	3	1	1.73300	52.781	10.0

32	1	2	3	1.72600	53.012	50.0
33	13	1	1	1.71600	53.345	40.0
34	11	2	1	1.66600	55.080	15.0
35	9	1	3	1.64800	55.733	70.0
36	14	1	1	1.61200	57.091	30.0
37	12	2	1	1.58300	58.236	45.0
38	7	3	1	1.56700	58.888	15.0
39	8	3	0	1.56000	59.179	20.0
40	6	0	4	1.55500	59.388	25.0
41	11	2	2	1.53000	60.459	20.0
42	8	3	2	1.41500	65.965	15.0
